# Characterization of a cryptic plasmid pSM429 and its application for heterologous expression in psychrophilic *Pseudoalteromonas*

**DOI:** 10.1186/1475-2859-10-30

**Published:** 2011-05-05

**Authors:** Dian-Li Zhao, Zi-Chao Yu, Ping-Yi Li, Zhao-Yu Wu, Xiu-Lan Chen, Mei Shi, Yong Yu, Bo Chen, Bai-Cheng Zhou, Yu-Zhong Zhang

**Affiliations:** 1 State Key Laboratory of Microbial Technology, Marine Biotechnology Research Center, Shandong University, Jinan 250100, China; 2 SOA Key Laboratory for Polar Science, Polar Research Institute of China, Shanghai 200136, China; 3 Jinan Zhi-Cheng Bio-Technology CO., LTD., Jinan 250100, China

## Abstract

**Background:**

*Pseudoalteromonas *is an important genus widespread in marine environment, and a lot of psychrophilic *Pseudoalteromonas *strains thrive in deep sea and polar sea. By now, there are only a few genetic systems for *Pseudoalteromonas *reported and no commercial *Pseudoalteromonas *genetic system is available, which impedes the study of *Pseudoalteromonas*, especially for psychrophilic strains. The aim of this study is to develop a heterologous expression system for psychrophilic *Pseudoalteromonas*.

**Results:**

A cryptic plasmid pSM429 isolated from psychrophilic *Pseudoalteromonas *sp. BSi20429 from the Arctic sea ice, was sequenced and characterized. The plasmid pSM429 is 3874 bp in length, with a G+C content of 28%. Four putative open reading frames (ORFs) were identified on pSM429. Based on homology, the ORF4 was predicted to encode a replication initiation (Rep) protein. A shuttle vector (*Escherichia coli, Pseudoalteromonas*), pWD, was constructed by ligating pSM429 and pUC19 and inserting a chloramphenicol acetyl transferase (CAT) cassette conferring chloramphenicol resistance. To determine the minimal replicon of pSM429 and to check the functionality of identified ORFs, various pWD derivatives were constructed. All derivatives except the two smallest ones were shown to allow replication in *Pseudoalteromonas *sp. SM20429, a plasmid-cured strain of *Pseudoalteromonas *sp. BSi20429, suggesting that the *orf4 *and its flanking intergenic regions are essential for plasmid replication. Although not essential, the sequence including some repeats between *orf1 *and *orf2 *plays important roles in segregational stability of the plasmid. With the aid of pWD-derived plasmid pWD2, the erythromycin resistance gene and the *cd *gene encoding the catalytic domain of a cold-adapted cellulase were successfully expressed in *Pseudoalteromonas *sp. SM20429.

**Conclusions:**

Plasmid pSM429 was isolated and characterized, and the regions essential for plasmid replication and stability were determined, helping the development of pSM429-based shuttle vectors. The shuttle vectors pWD and its derivatives could be used as cloning vectors for *Pseudoalteromonas*, offering new perspectives in the genetic manipulation of *Pseudoalteromonas *strains. With the aid of pWD-derived vector and its host, the erythromycin resistance gene and the *cd *gene of a cold-adapted protein were successfully expressed, indicating that the potential use of this system for recombinant protein production, especially for cold-adapted proteins.

## Introduction

Cold-adapted bacteria are excellent candidates for investigations of protein evolution and molecular adaptations to extreme conditions. Moreover, cold-adapted enzymes from psychrophiles offer novel opportunities for biotechnological applications [1,2]. Native plasmids from cold-adapted bacteria are of particular interest, because plasmids contribute directly in the adaptation of bacteria to their natural environments and provide easy model systems for investigation of basic molecular processes [3]. Furthermore, native plasmids can be used in developing genetic systems.

*Pseudoalteromonas *is a genus of gamma-proteobacteria that is widespread in the world's oceans, and a lot of psychrophilic *Pseudoalteromonas *strains thrive in deep sea and polar sea. The genus *Pseudoalteromonas *contains over 30 marine species [4], which play an important role in marine ecosystem. *Pseudoalteromonas *has attracted significant interest for two reasons. First, *Pseudoalteromonas *species are important for investigation of microbe-host interactions in the sea. Second, many species synthesize biologically active molecules, such as extracellular antibiotics, toxins, extracellular enzymes and polysaccarides [4-8]. However, by now there are only a few genetic systems for *Pseudoalteromonas *reported [9-12] and no commercial *Pseudoalteromonas *genetic system is available, which impedes the study of *Pseudoalteromonas*, especially for psychrophilic strains.

In our laboratory, psychrophilic *Pseudoalteromonas *strains have been studied for over ten years on their cold-adaptation mechanism and the proteases they secreted [5,13-18]. To further study the cold-adaptation mechanism of psychrophilic *Pseudoalteromonas *and protease expression, development of genetic systems for gene knockout and cold-adapted protein expression is essential. In this article, a small cryptic plasmid from psychrophilic *Pseudoalteromonas *sp. BSi20429 (BSi20429 thereafter), designated pSM429, was isolated and characterized. A shuttle vector pWD was constructed based on pSM429. The regions responsible for replication and stability on pWD were determined by studying the replication of pWD-derived plasmids in *Pseudoalteromonas *sp. SM20429 (SM20429 thereafter), a plasmid-cured strain of BSi20429. A stable pWD-derived plasmid, pWD2, was successfully used as an express vector to express the erythromycin resistance gene and the *cd *gene of a cold-adapted cellulase in SM20429.

## Materials and methods

### Bacterial strains and plasmid isolation

*Pseudoalteromonas *strains were previously isolated from the sea-ice samples collected using a MARKII ice auger during the Second Chinese National Arctic Research Expedition cruise of the USCGC icebreaker Xue Long into the Canada Basin in August 2003. 102 *Pseudoalteromonas *strains were screened for the presence of plasmids. Alkaline SDS lysis and DNA preparation method was used to isolate plasmids as described by Sambrook et al. [19]. *Pseudoalteromonas *sp. BSi20429 grown at 20°C in 2216E broth was found to carry a plasmid. Strain BSi20429, a gram negative bacterium, grows well in aerobic conditions at temperatures from 4°C to 34°C in 2216E broth (Difco) or marine LB broth (10 g peptone, 5 g yeast extract, 1 l artificial seawater, pH 7.5). The optimal temperature for BSi20429 growing is 20-25°C. *Escherichia coli *DH5α [(*supE *44, △*lac*U169 (θ80*lacZ*△*M15*) *endA1, recA1, hsdR17, thi-1 *l- *gyrA96, relA1*] was grown at 37°C in LB medium [19] and used as a host for gene cloning and plasmid construction. *Pseudoalteromonas *transformants were selected in the presence of 8 μg/ml chloramphenicol and 25 μg/ml ampicillin, and further propagated in broth medium containing 20 μg/ml choramphenicol. Antibiotics were used in *E. coli *at the following concentrations: ampicillin, 100 μg/ml; chloramphenicol, 25 μg/ml. Plasmids used in this study were listed in Table 1.

**Table 1:** Plasmids used in this study

Plasmids	Relevant characteristics	Source or reference
pGEM-T easy	Cloning vector, Apr	Promaga
pMD19T simple	Cloning vector, Apr	Takara
pMG36e	Erythromycin^r ^gene source for vector construction	[17]
pBT	CAT cassette source for vector construction	Stratagene
pUC19	Cloning vector, Apr	Takara
pSM429	Cryptic plasmid from *P*. sp. BSi20429	This study
pWD	*E. coli-Pseudoalteromonas *shuttle vector	This study
pWD1	pWD derivative, 1500-bp fragment deleted from pWD	This study
pWD2	pWD derivative, 250-bp fragment deleted from pWD1	This study
pWD3	pWD derivative, 150-bp fragment deleted from pWD2	This study
pWD4	pWD derivative, 320-bp fragment deleted from pWD3	This study
pWD5	pWD derivative, 280-bp fragment downstream deleted from pWD4	This study
pWD6	pWD derivative, 140-bp fragment upstream deleted from pWD4	This study
pWD-ermC	pWD2-derived *ermC *expression vector, *Plac-ermC *fusions	This study
pWD-cd	pWD2-derived *cd *expression vector, *Plac-cd *fusions	This study

### Plasmid sequencing and analysis

The plasmid isolated from BSi20429 was termed as pSM429. Analysis of restriction digests on agarose gel showed that pSM429 contains singular restriction site for *Hin*dIII and *Nde*I, respectively. The plasmid was linearised by these two restriction enzymes, and restriction fragments of different sizes were cloned into pGEM-T Easy and sequenced by dideoxy chain termination method using an Applied Biosystems DNA sequencer. The nucleotide sequence of pSM429 was determined by using a combination of subcloning and primer walking. The whole plasmid sequence was covered at least three times by single read. The nucleotide sequence, amino acid sequence and the distribution of direct and inverted repeated sequences were analyzed using DNASTAR. The predicted protein-coding regions were initially defined by searching for ORFs longer than 80 codons. The potential coding regions were then confirmed with prokaryotic gene finder GeneMark.hmm 2.4 for Prokaryotes [20] at http://opal.biology.gatech.edu/GeneMark/gmhmm2_prok.cgi using *Pseudoalteromonas haloplanktis *TAC125 chromosome I genome as a model. The functional protein domain analysis of predicted ORFs was performed by the InterProScan program at the European Bioinformatics Institute http://www.ebi.ac.uk/InterProScan/. Similarity searches were carried out using Blast http://www.ncbi.nlm.nih.gov/blast/[21].

The nucleotide sequence of pSM429 was deposited in the GenBank database under the accession number EU627679.

### Determination of plasmid copy number

The copy number of plasmid pSM429 in strain BSi20429 was determined using the method described by Qin et al. [22]. Strain BSi20429 was grown in marine LB broth at 20°C and total genome DNA was isolated by using PowerMax™Soil DNA Isolation Kit (MO BIO). The genome DNA was diluted by 10 and 100 times in TE Buffer (10 mM Tris-HCl, 1 mM EDTA, pH 8.5), and then was separated in equal volumes on a 0.7% agarose gel by electrophoresis at 50 V for 2.5 h. By comparing the intensity of the diluted chromosomal DNA band with the non-diluted plasmid DNA band using the image analysis software Gene Tools (Syngene), the copy number of pSM429 was calculated as follows:

(*N_c_*, copy number of the plasmid; *S_c_*, chromosomal DNA size (4.5 M); *S_p_*, plasmid DNA size (3.78 kb); *I_p_*, intensity of plasmid DNA band; *I_c_*, intensity of chromosomal DNA band).

### Curing of pSM429 from BSi20429

BSi20429 was grown for at least 8 subcultures (approximately 160 generations) in 2216E broth containing 0.07% SDS. An aliquot of culture was serially diluted and spread onto 2216E agar. Colonies were screened for the presence of pSM429 as described above. Putative cured isolates were checked for plasmid integration into the genome by PCR with two pairs of primers covering different regions of pSM429 (RepF & RepR, ORF3F & ORF3R). Primer sequences used in this study were shown in Table 2.

**Table 2:** Pimers used in this study

Name	Sequence (5'-3') *
RepF	CACAAAACGCCCTACAA
RepR	GACCCGACAAATGATT
ORF3F	ACTTACATTGTCATCC
ORF3R	CCTTCACAGTGTCTTG
CB	CGCGGATCCACGCACCACCCCGTCAGTAG (BamHI)
CE	CCGGAATTCGAAGCACACGGTCACACTG (EcoRI)
EMF	AACTGCAGCTAATTTTATAAGGAG (PstI)
EMR	ACGCGTCGACGTTAAGGGATGCAG (SalI)
AF	ACGCGTCGACTTTACCCTTACGCATAC (SalI)
AR	CGGGATCCTTACGCTTCGCTTGTCTG (BamHI)
BF	ACGCGTCGACAAGACACTGTGAAGGC (SalI)
BR	CGGGATCCTGCCTTTAAGATTTGC (BamHI)
CR	CGGGATCCAGTCATTCTTGATTAAGTAC (BamHI)
DR	CGGGATCCTTTCATTATTGCCTCG(BamHI)
ER	CGGGATCCCTGATACCACCATACTG(BamHI)
CF	ACGCGTCGACGAATGGTTAGCCCTTAAG (SalI)
PucF	CGGAATTCGGAGCTGCATGTGTC (EcoRI)
PucR	ACGCGTCGACTCTTCCGCTTCCTC (SalI)

*The restriction enzyme sites in the primers were underlined, and the corresponding restriction enzymes were shown in brackets.

### Construction of the shuttle vector pWD and its derivatives

Plasmids pSM429 and pUC19 were isolated as described above. The complete DNA sequence of pSM429 was amplified by PCR with primers AF (containing a *Sal*I site) and AR (containing a *Bam*HI site). The 3.8-kb linearised pSM429 was digested with *Sal*I and *Bam*HI and cloned in pUC19. The chloramphenicol acetyl transferase (CAT) cassette was amplified by PCR from pBT (Stratagene), using primers CB (containing a *Bam*HI site) and CE (containing an *Eco*RI site). The 1.3-kb product was double-digested using *Bam*HI and *Eco*RI and ligated to the constructed pUC19+pSM429 treated with the same restriction enzymes, and then transformed into *E. coli *DH5α. The constructed shuttle vector was designated as pWD.

**Figure 1:**
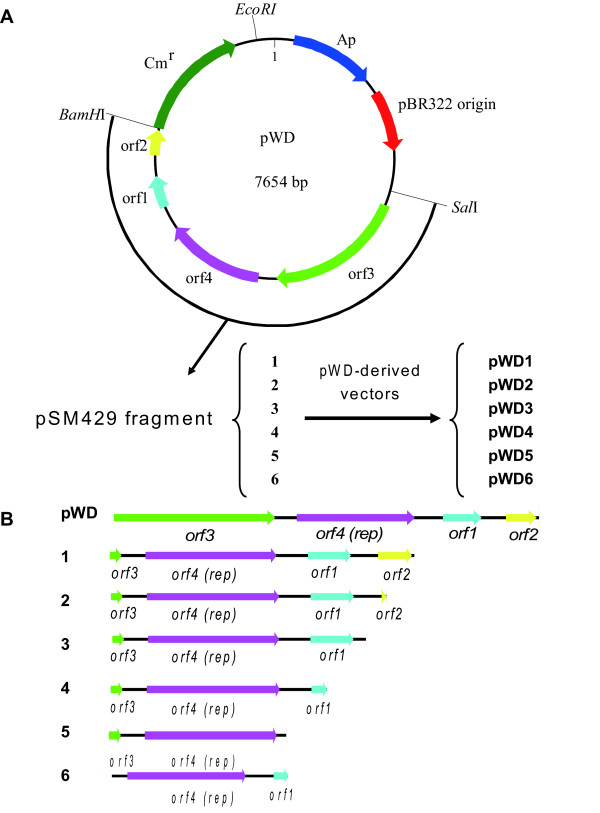
**Map of shuttle vector pWD (A) and schematic diagram of the pWD derivatives (B)**. *Sal*I and *Bam*HI sites were introduced with the oligonucleotide primers used for amplification. Primers BF/AR, BF/BR, BF/CR, BF/DR, BF/ER and CF/DR were used to amplify pSM429 derivatives pWD 1-6, respectively. Arrows denote direction and approximate length of the different ORFs.

Derivatives of pWD (pWD1, pWD2, pWD3, pWD4, pWD5, pWD6) were constructed as outlined in Figure 1. Purified DNA of pSM429 was used as a template in PCR. All primers were designed with sites for *Sal*I or *Bam*HI at their 5' ends to allow direct cloning of the amplicons digested with these two enzymes into the *Sal*I and *Bam*HI sites of pWD. In new constructs, the complete fragment of pSM429 was replaced by the amplicons with different length. New constructed derivatives were transformed into *E. coli *DH5α. Plasmids isolated from *E. coli *DH5α and verified by sequencing were electroporated into SM20429.

### Electroporation

The protocol to electroporate plasmids into SM20429 was based on the method described by Kurusu et al. [23] with some modification. A 1% inoculum from over-night culture of SM20429 in 2216E medium was inoculated into 20 ml of the same culture and grown at 20°C. After incubation for 4 h, the logarithmic-phase cells were harvested and washed twice with chilled ddH_2_O. The cells were suspended in a final volume of 100 μl ddH_2_O containing 20 mM glucose, and mixed with 1 μg plasmid DNA. Electroporation was performed with a Gene Pulser (Bio-Rad) at a capacity of 50 μF and a voltage of 2.0 kV. After one pulse, the cells were grown in 2216E medium supplemented with glucose (20 mM) for 3 h, and then were spread on selective plates with chloramphenicol.

### Analysis of plasmid stability

SM20429 strains with plasmid pWD or other derivative plasmids were grown in 2216E broth for about 24 generations by being serially transferred to fresh broth medium. Samples were removed from each transfer and serial dilutions of the sample were plated on 2216E agar medium with or without antibiotics. The fraction of chloramphenicol-resistant (CmR) cells in the total population was determined to calculate the segregational stability of the constructed plasmid vectors. Finally, presence of plasmid DNA in CmR cells was confirmed by plasmid isolation and transformation to *E. coli *DH5α.

### Construction of expression system in SM20429

The erythromycin resistance gene (*ermC*) from plasmid pMG36e [24] was amplified by PCR using the primers EMF (containing *Pst*I restriction site) and EMR (containing *Sal*I restriction site) to construct an *ermC*-expression vector. The 735-bp PCR product was cloned into vector pMD19T simple (Takara). The fragment of the promoter-less *ermC *gene in the pMD19T was separated with *Pst*I and *Sal*I and was used in the construction of the *ermC*-expression vector with the smallest stable plasmid pWD2.

The promoter-less erythromycin resistance gene was cloned into pWD2 downstream of the *Plac *of pUC19. The constructed *ermC*-expression vector was designated as pWD-ermC. This expression vector was electroporated into SM20429. The transformed SM20429 cells were cultured at 25°C in 2216E broth for 24 h. To test the gene expression in SM20429, the total cell lysates of recombinant strain SM20429 expressing *ermC *under the control of *Plac *were analyzed by 12.5% sodium dodecyl sulfate-polyacrylamide gel electrophoresis (SDS-PAGE). To test whether the recombinant ErmC was active, the transformed SM20429 cells harboring pWD2 and the transformed SM20429 cells harboring pWD-ermC were inoculated in 2216E medium supplemented with 30 μg/ml or 60 μg/ml erythromycin (EM), respectively, and then cultured at 25°C.

### Gene cloning and expression of the catalytic domain of the cold-adapted cellulase from *P*. sp. BSw20308 and cellulase assay

A gene encoding a cold-adapted cellulase Cel308 (GenBank Accession No. HQ997897) has been cloned from the psychrophilic strain *Pseudoalteromonas *sp. BSw20308 isolated from the Arctic sea ice in our lab. The DNA fragment encoding the catalytic domain (CD) of the cellulase was amplified by PCR from BSw20308, and then ligated into pWD2 to construct the expression vector pWD-cd. The vector pWD-cd was introduced into SM20429 by electroporation for the production of the CD. The transformed SM20429 cells were cultured at 25-30°C in 2216E broth. To analyze the expression of the CD of the cellulase, the cellulase activity in the transformed SM20429 cells was assayed. After grown at 25-30°C for 48 h, the SM20429 cells (3 ml) were harvested and resuspended in 5 ml PBS buffer (0.02 M, pH 7.5), and then sonicated in an ice-water bath. After centrifugation, the cellulase activity in the supernatant was measured as described by Bhat et al. [25]. A mixture containing 100 μl of the supernatant and 900 μl of 1% sodium carboxyl methyl cellulose was incubated at 35°C for 30 min. After incubation, the released amount of reducing sugar was measured using the dinitrosalicylic acid (DNS) method with glucose as standard [26]. One unit of the enzyme activity was defined as the amount of enzyme to release 1 μg of glucose per min.

## Results

### Characterization of the cryptic plasmid from BSi20429

A cryptic plasmid, designed as pSM429, was isolated from *Pseudoalteromonas *sp. BSi20429. The copy number of pSM429 in BSi20429 was about 30 per cell (Additional file 1, Figure S1). The complete sequence of pSM429 consists of 3874 bp with a G+C content of 28%, which is lower than that of the chromosomal DNA (39-44%) of *Pseudoalteromonas*, as well as that of other published *Pseudoalteromonas *plasmids, such as pPS1M3 (37%) [23], pMtBL (39%) [27] and pKW1 (43%) [28]. Sequence analysis revealed that the plasmid pSM429 contains four open reading frames (ORFs) with more than 80 amino acid residues (Table 3).

**Table 3:** ORF analysis of pSM429 from *Pseudoalteromonas *sp. BSi20429

ORF	Function	Position (size)^a^	MW^b^ (Da)	Identity (/) ^c^	Best BLAST Match	GenBank Accession No.
ORF1	Unknown	159-488 (109)	13011	37% (44/110)	Hypothetical protein of *Pseudomonas syringae*	ZP 03395526
ORF2	Unknown	697-957 (86)	9759	57% (47/83)	Hypothetical protein of *Aliivibrio salmonicida*	YP 002264558
ORF3	Unknown	1195-2604 (469)	55068	26% (94/371)	Hypothetical protein of *Vibrio parahaemolyticus*	NP 798516
ORF4	Replicase	2789-3793 (334)	38728	28% (84/306)	Replication protein A of *Salmonella enterica*	YP 002045467

*a *Number of amino acids.

*b *Predicted molecular weight.

*c *The number of identical amino acids/the number of total amino acids in the region of homology identified by BLAST.

**Figure 2:**
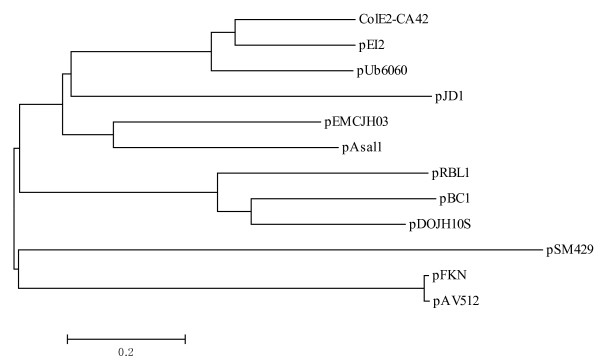
**Phylogenetic analysis of the Rep proteins from pSM429 and related plasmids**. The unrooted phylogenetic tree was constructed by the sequence distance method using the neighbor-joining algorithm. The GenBank accession numbers of the Rep proteins are as follows: ColE2-CA42 from *Escherichia coli*, BAA0694; pEI2 from *Edwardsiella ictaluri*, NP_061811; pUb6060 from *Plesiomonas shigelloides*, CAB56518; pEMCJH03 from *Moraxella catarrhalis*, NP_957539; pAsal1 from *Aeromonas salmonicida*, NP_710167; pAV512 from *Pseudomonas syringae*, AAZ29772; pBC1 from *Bifidobacterium catenulatum*, YP_241107; pDOJH10s from *Bifidobacterium longum*, NP_694604; pRBL1 from *Brevibacterium linens*, AAB03568; pJD1 from *Neisseria gonorrhoeae*, NC_040411.

Searches in the databases revealed that ORF4 showed moderate similarity (25-26% identity, 50% similarity) with the Rep proteins of pFKN [29] and the other plasmids belonging to pPT23A family from *Pseudomonas syringae *[30,31]. It also showed some similarity (with identity lower than 26%) with the Rep proteins of the ColE2-related plasmids, as well as the Rep proteins of plasmids isolated from the bacteria of different genus. As plasmids are generally classified based on the amino acid sequence similarity between their replication initiator proteins [32], a phylogenetic tree was constructed using Rep amino acid sequences of some related plasmids as shown in Figure 2. pFKN and pAV505 isolated from *Pseudomonas syringae *were selected from pPT23A group and the other plasmids were found by homology search using putative Rep amino acid sequence of pSM429. All of the 12 Rep proteins in the phylogenetic tree contain two conserved domains [33], a replicase domain (Pfam Accession No. PF03090) believed to be involved in DNA binding [34], and an alpha helical domain (Pfam Accession No. PF08708) that was found at the C terminus of primases [35]. This suggests that these Rep proteins might be involved in the replication with the similar mechanism. However, pSM429 did not cluster with other plasmids in the phylogenetic tree, implying that pSM429 is a novel plasmid.

### Construction of the shuttle vector pWD and transformation system

The shuttle vector pWD, based on plasmid pSM429, was constructed for replication in *E. coli *and *Pseudoalteromonas *strains (Figure 1). The 3.8-kb plasmid pSM429 was linearised and cloned in pUC19. In addition, the 1.3-kb chloramphenicol resistance gene was inserted into the resulted plasmid as selection marker to construct the shuttle vector pWD. The presence of the pUC19 ori and pSM429 ori in pWD enabled pWD to replicate in both *E. coli *and BSi20429. The constructed plasmid pWD could be purified from *E. coli *in a large amount.

To construct the host strain for the plasmid transformation, the plasmid pSM429 was cured from BSi20429 using the SDS-treated method. Screening of 40 colonies yielded 4 plasmid-cured colonies, showing an efficiency of 10%, which is much more effective than the method used in *Pseudoalteromonas *strains previously reported [23]. One plasmid-cured strain showing a similar phenotype to the parental strain was designated as SM20429, which was used for further study. The total DNA of SM20429 was purified and used as template for PCR checking. The results showed that the plasmid did not integrate into the genome of SM20429. As SM20429 was sensitive to erythromycin and chloramphenicol (data not shown), chloramphenicol was used for the direct selection of *Pseudoaltermonas *transformants. The constructed shuttle vector pWD was introduced into SM20429 by electroporation. Chloramphenicol-resistant transformants of SM20429 were obtained at the frequency of 5.0x 10^2^/μg DNA. To confirm that the *Pseudoalteromonas *transformants harbored plasmids, several transformants were subjected to plasmid analysis. Electrophoresis showed that plasmids were prepared from all of the selected transformants, and the prepared plasmids showed the same electroporetic DNA pattern as that of pWD (Additional file 2, Figure S2). Moreover, pWD DNA prepared from the *Pseudoalteromonas *transformants could be transformed into SM20429 at a higher frequency (10^4^/μg DNA) than that prepared from *E. coli *transformants, suggesting the presence of DNA restriction and modification system in BSi20429.

### Properties of the shuttle vector pWD and its derivatives

The shuttle vector pWD constructed above harbors the complete sequence of plasmid pSM429. To check the functionality of the identified ORFs on pSM429 and delete the unnecessary regions on pWD for an expression vector, six plasmids derived from pWD with different deletions were constructed (Figure 1). Each of the six derivatives was checked in *E. coli*, and then was introduced into SM20429 by electroporation. Except for the two smallest derivatives, pWD5 and pWD6, transformants of the other four derived plasmids were all obtained, indicating that pWD5 and pWD6 were not capable of replication in SM20429. Since pWD5 and pWD6 lack the upstream or the downstream sequences of the *rep *gene (*orf4*) compared to pWD4, the results indicated that the fragment containing *rep *gene and its flanking sequences are the minimal replication region of pSM429 and all of *orf1, orf2 *and *orf3 *are not essential for plasmid replication.

**Figure 3:**
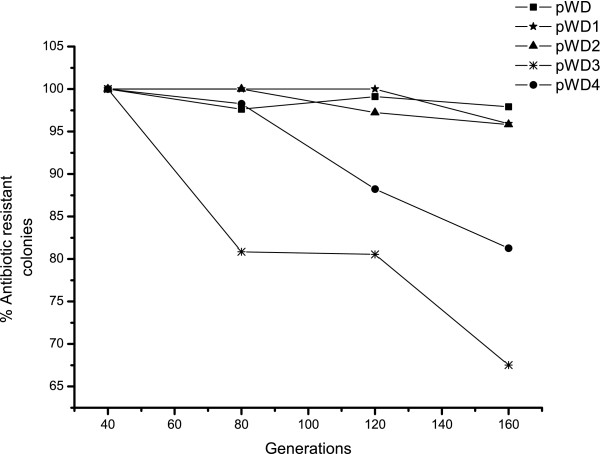
**Segregational stability of pWD and its derivatives in SM20429**. Average results of two independent experiments for each construct were presented. Strains harboring the constructs were cultured in the absence of selective pressure, plated under the same conditions, and assayed for plasmid maintenance by replica-plating onto antibiotic-containing media at 40-, 80-, 120-, and 160-generation intervals. The presence of plasmids was finally checked by plasmid preparation and gel electrophoresis.

**Figure 4:**
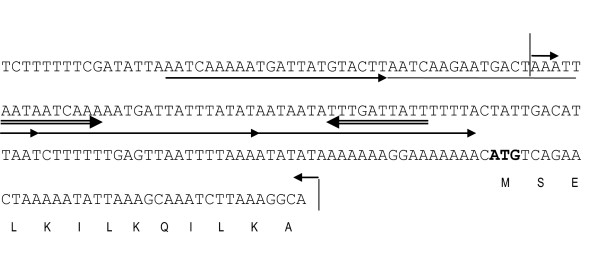
**Nucleotide sequence of the 200-bp region between *orf1 *and *orf2 *in pSM429 related to plasmid segregational stability**. The region lacking in pWD3 compared to pWD2 is shown between two vertical lines. The four imperfect direct repeats are marked with single arrows, and the inverted repeat is marked with double arrows. The deduced amino acid sequence of ORF2 is shown and the initiation coden is shown in bold.

The segregational stability of pWD and its four derivatives were tested twice and counts were done in duplicate. Average results were presented in Figure 3. In the absence of selective pressure, nearly 100% of the cells retain plasmids in the first 40 generations of SM20429. However, after 40 generations, the smaller derivatives, pWD3 and pWD4, exhibited less segregational stability than pWD and the other bigger derivatives, pWD1 and pWD2, in SM20429. Compared to pWD2, pWD3 lacks some repeat sequences located between *orf1 *and *orf2*, as shown in Figure 4. The above result suggests that these repeat sequences are essential for the segregational stability of the plasmid and possibly related to plasmid partition.

### Construction of an *ermC*-expression vector and expression of ErmC in SM20429

**Figure 5:**
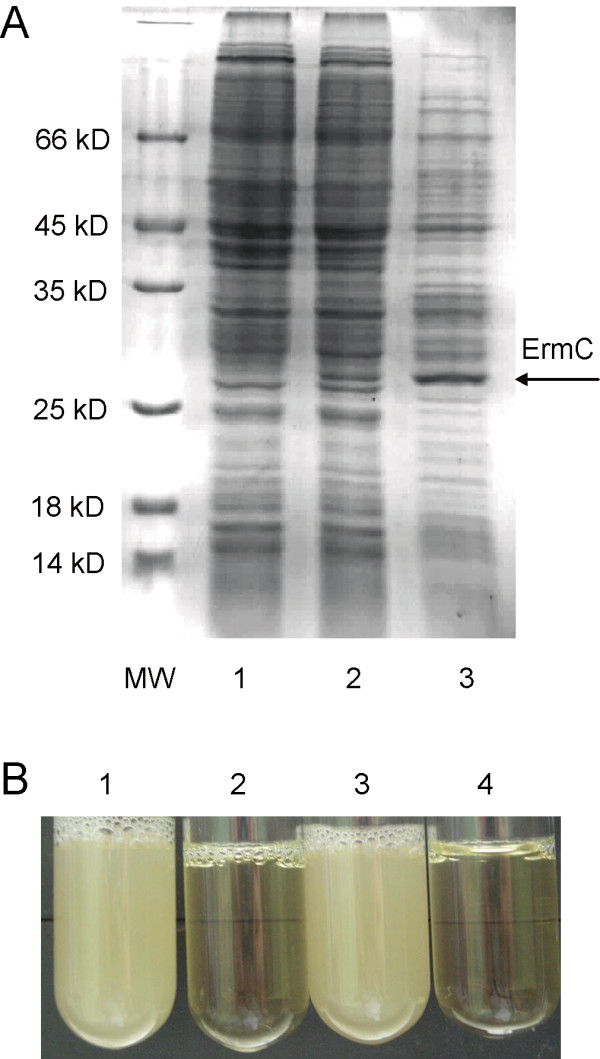
**Expression of the recombinant ErmC protein in SM20429 and *E. coli *with the constructed vector pWD-ermC**. (A) SDS-PAGE analysis of the expressed ErmC protein in the total-cell lysates of recombinant SM20429 and *E. coli*. Lane 1, SM20429 harboring pWD2, used as a negative control; Lane2, SM20429 harboring pWD-ermC; Lane3, *E. coli *DH5α harboring pWD-ermC, used as a positive control. The SDS-PAGE gel was stained with Coomassie blue. Arrow denotes the band of the expressed ErmC. (B) Analysis of the ErmC-resistance of the transformed SM20429. 1, pWD-ermC-harboring SM20429 inoculated in 2216E medium supplemented with 30 μg/ml EM; 2, pWD2-harboring SM20429 inoculated in 2216E medium supplemented with 30 μg/ml EM; 3, pWD-ermC-harboring SM20429 inoculated in 2216E medium supplemented with 60 μg/ml EM; 4, pWD2-harboring SM20429 inoculated in 2216E medium supplemented with 60 μg/ml.

The erythromycin resistance gene *ermC *was cloned into pWD2 downstream of the *Plac *promoter to construct an *ermC*-expression vector, pWD-ermC. This vector was introduced into SM20429 by electroporation and the *ermC *gene was expressed under the control of *Plac *promoter. SDS-PAGE analysis showed that the ErmC protein was expressed in SM20429 cells, and the expression level of ErmC in SM20429 was lower than that in *E. coli *DH5α (Figure 5A). To test whether the expressed ErmC protein is active, the SM20429 strain harboring pWD2 and the SM20429 strain harboring pWD-ermC were inoculated in 2216E medium supplemented with erythromycin (EM), respectively. The pWD2-harboring strain that is sensitive to EM as the wild strain BSi20429 could not grow in the medium supplemented with 30 μg/ml EM, while the pWD-ermC-harboring strain grew well in the medium supplemented with 60 μg/ml EM (Figure 5B). Therefore, the pWD-ermC-harboring strain obtained the ability resistant to EM, indicating that the ErmC protein expressed in SM20429 was active. These results suggest that *Plac *promoter is efficient for heterologous gene expression in SM20429.

### Expression of cold-adapted protein with the constructed expression system

**Figure 6:**
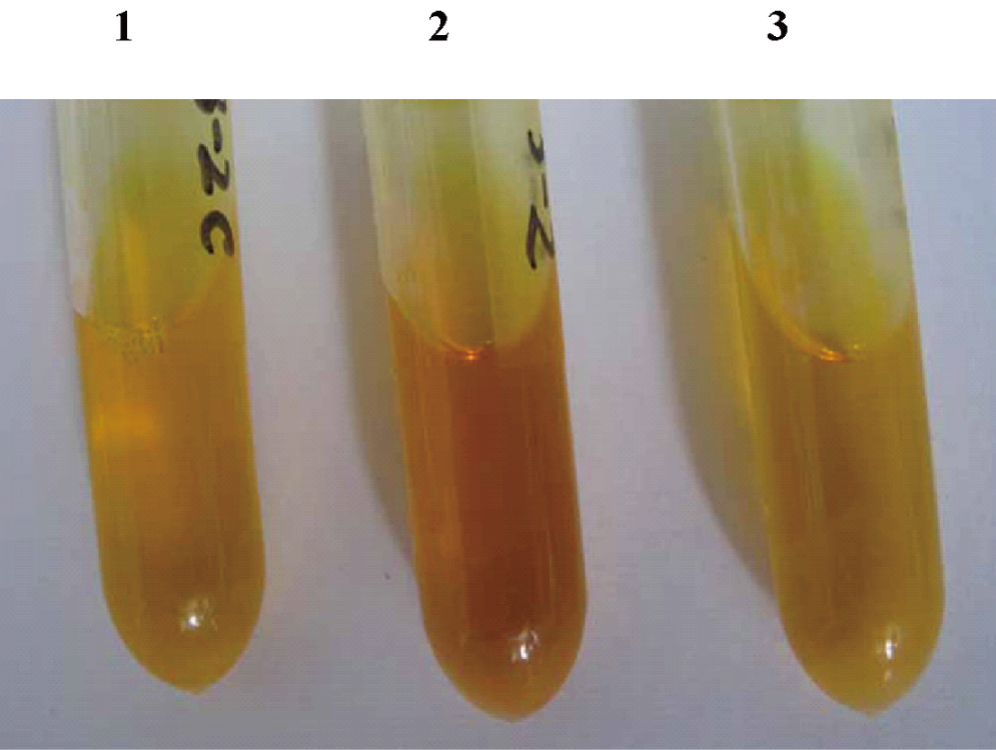
**Assay of the intracellular cellulase activity of the transformed SM20429**. 1, Control: DNS was added before enzymatic reaction starting; 2, SM20429 harboring pWD-cd; 3, SM20429 harboring pWD2.

In our previous work, a cellulase named Cel308 was purified from the Arctic sea ice bacterium *Pseudoalteromonas *sp. BSw 20308 (Additional file 3, Figure S3), and the gene encoding Cel308 was cloned and sequenced. Character analysis showed that Cel308 is a cold-adapted enzyme (Additional file 3, Figure S4). The *cd *gene encoding the catalytic domain of Cel308 was cloned into pWD2 to construct a *cd*-expression vector pWD-cd. The plasmid pWD-cd was transformed into SM20429 by electroporation. After cultivation for 48 h, the intracellular cellulase activity of the transformed strain was detected using a quantitative assay. The results showed that 60 units of cellulase enzyme were expressed in 1 ml culture of SM20429. In comparison, no cellulase activity was detected in the cells of SM20429 harboring pWD2 (Figure 6). The results indicated that the constructed expression system consisting of strain SM20429 and vector pWD2 could be used to express cold-adapted protein.

## Discussion

As the first step to develop an effective genetic system for *Pseudoalteromonas *strains, especially the psychrophilic ones, the *Pseudoalteromonas *strains isolated from the Arctic sea ice were screened for plasmids. Only one small plasmid, pSM429, was obtained out of over one hundred strains, suggesting that small plasmids from the Arctic sea ice isolates of *Pseudoalteromonas *are less common. This finding is consistent with the fact that only a few reports have been published about the isolation and characterization of plasmids from cold-adapted *Pseudoalteromonas *strains, including *Pseudoalteromonas *sp. PS1M3 [23], *Pseudoalteromonas haloplanktis *TAC125 [27] and *Pseudoalteromonas *sp. 643A [36]. pSM429 has much lower G+C content than other *Pseudoalteromonas *plasmids, implying its cold-adaptation. The small plasmid pSM429 is valuable for expanding the knowledge about the genetics of extrachromosomal elements in cold-adapted *Pseudoalteromonas *strains and opens the possibility to construct a genetic system for *Pseudoalteromonas *strains.

Genetic systems require stable cloning and expression vectors that allow efficient cloning and expression of homologous and heterologous genes. To develop a genetic system of psychrophlic *Pseudoalteromonas*, a plasmid-cured strain of BSi20429 was obtained to be used as an expression host, and a shuttle vector for *Pseudoalteromonas-E. coli *was constructed based on pSM429. Sequential deletions were used to study the functionality of ORFs in plasmid pSM429 and to analyze their effect on plasmid stability. The results showed that the *rep *gene is essential for the autonomous replication of pSM429 in SM20429. This is consistent with the finding that plasmids need specific replication protein to recognize and cut the double-stranded origin of replication for the process to begin [37]. Furthermore, deletion of surrounding sequences of *rep *gene caused the loss of replication ability of the plasmid in SM20429, probably because these regions contain the promoter sequence of *rep *gene and the replication origin where the Rep protein binds. Although not essential, an intergenic fragment including some repeat sequences on pSM429 markedly affects the segregational stability. Accordingly, the stable construction pWD2 containing the replication region and the intergenic region related to segregational stability was used as an *E. coli*- *Pseudoalteromonas *shuttle vector and for construction of expression vectors.

An expression system in cold-adapted bacteria may circumvent the problem of limited stability of the product that is always encountered during heterogonous expression of psychrophilic proteins in mesophilic hosts [15,38]. A few low-temperature expression systems have been reported [39,40]. However, there are still many cold-adapted proteins could not be successfully expressed by a recombinant protein expression system. The alternative systems would be important for the study of cold-adapted proteins. In order to check the usefulness of plasmid pWD2 and its host SM20429 as expression system, the promoter-less *ermC *gene was cloned into the vector pWD2 and introduced into SM20429. The results showed that the *ermC *gene was expressed in SM20429 and the recombinant strain displayed resistance to erythromycin, suggesting that the *Plac *promoter was efficient in SM20429. Then, with this system, the *cd *gene encoding the catalytic domain of a cold-adapted cellulase from a psychrophilic strain was successfully expressed, suggesting the potential use of this system for overexpression of genes from psychrophiles. Indeed, this low-temperature expression system is still in an early stage of development. Further improvement, such as increasing the yield of proteins and controlling the expression of proteins, is being underway.

## Conclusion

In this paper, we report the characterization and use of a plasmid pSM429 from the Arctic *Pseudoalteromonas *strain BSi20429. The plasmid pSM429 is 3874 bp in length, with a very low G+C content (28%). The ORF4 on pSM429 was predicted to encode a Rep protein. Phylogenetic analysis implied that pSM429 is a novel plasmid. The regions essential for plasmid replication and stability were determined. Accordingly, the stable shuttle vectors based on pSM429 for *E. coli *and *Pseudoalteromonas *strains, pWD, pWD1 and pWD2, were developed, which can be used as cloning vectors for the *Pseudoalteromonas *strain we studied. With the aid of pWD2 and its *Pseudoalteromonas *host, the heterologous expressions of the erythromycin resistance gene and the cold-adapted cellulase gene were successful, indicating the potential use of this system for recombinant protein production, especially for cold-adapted proteins. These results are helpful for *Pseudoalteromonas *study.

## Competing interests

The authors declare that they have no competing interests.

## Authors' contributions

XC and YZ designed the project; YY and BC provided the strains; DZ, ZY, PL and ZW performed the research; DZ. ZY, XC, MS, BZ and YZ analyzed the data; DZ wrote the paper; YZ and XC critically reviewed the paper. All authors approved the final manuscript.

## Supplementary Material

Additional file 1 **Figure S1: Agrose gel electrophoresis analysis of total genominc DNA from BSi20429**. Genomic DNA, including the chromosome and plasmid, was isolated and then diluted by 10 and 100 times and electrophoresed in a 0.7% agarose gel. M, Trans15K DNA ladder (TransGen Biotech); Genomic DNA in lane 1 to lane3 were non-diluted, 10-time diluted and 100-time diluted. The open circular (OC) and covalently closed circular (CCC) forms of pSM429 are indicated. Click here for file

Additional file 2 **Figure S2: Agrose gel electrophoresis of plasmid pWD**. Lanes 1, 2 and 3 show the plasmid pWD isolated from transformed SM20429; Lane 4 shows the original plasmid pWD isolated from transformed *E. coli*. Click here for file

Additional file 3 **Characteristics of the cellulase Cel308 from the psychrophilic bacterium *Pseudoalteromonas *BSw20308**. Purification, characterization and gene cloning of the cold-adapted cellulase Cel308 from the psychrophilic bacterium *Pseudoalteromonas *sp. BSw 20308. It contains Figure S3 and Figure S4. Click here for file
