# Supplementary figures and images for: Characterization of a cryptic plasmid pSM429 and its application for heterologous expression in psychrophilic Pseudoalteromonas
Source: Microb Cell Fact. 2011 May 5;10:30. doi: 10.1186/1475-2859-10-30 (PMC3112385; doi:10.1186/1475-2859-10-30)

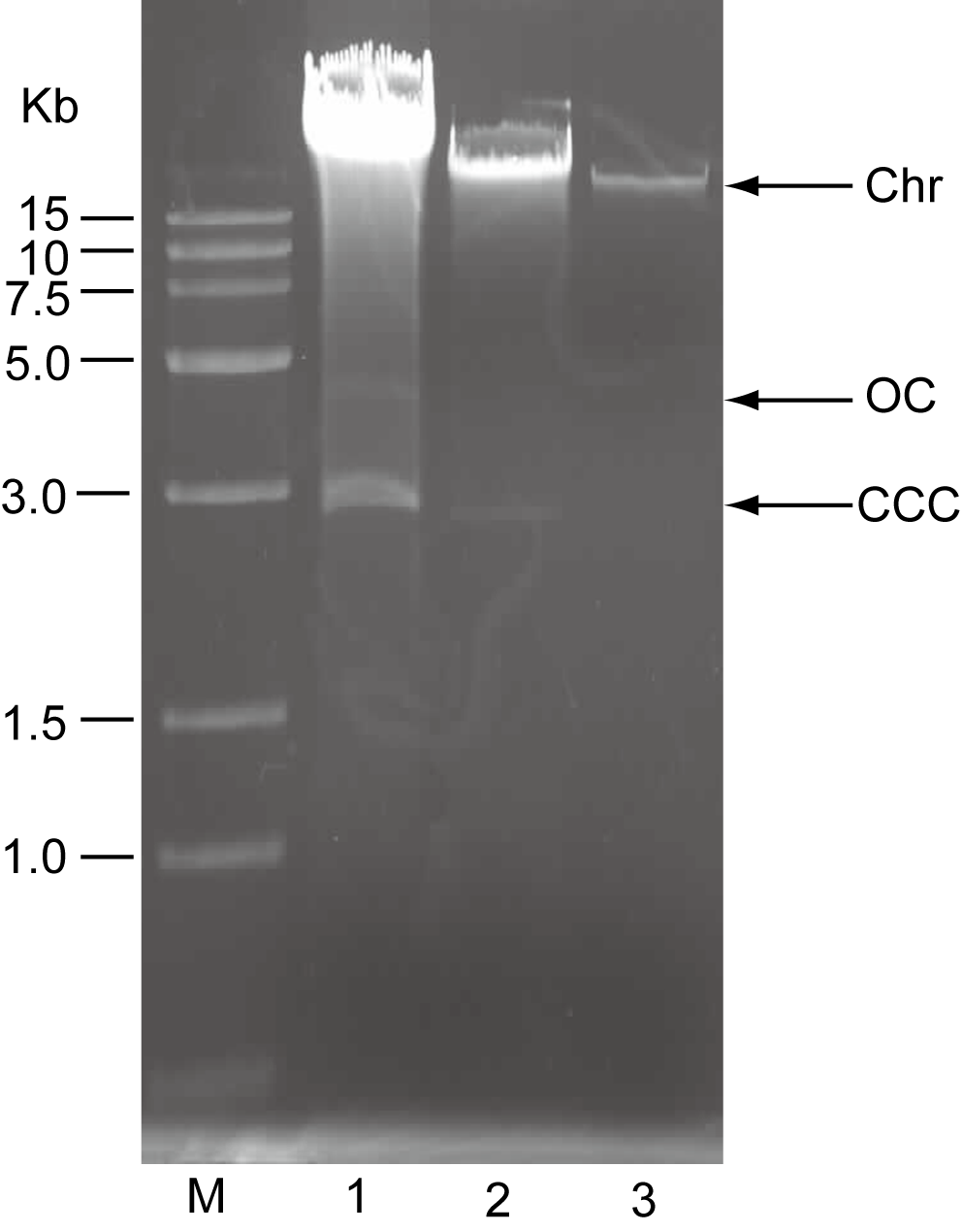

Supplement: Additional file 1 — Figure S1: Agrose gel electrophoresis analysis of total genominc DNA from BSi20429. Genomic DNA, including the chromosome and plasmid, was isolated and then diluted by 10 and 100 times and electrophoresed in a 0.7% agarose gel. M, Trans15K DNA ladder (TransGen Biotech); Genomic DNA in lane 1 to lane3 were non-diluted, 10-time diluted and 100-time diluted. The open circular (OC) and covalently closed circular (CCC) forms of pSM429 are indicated. [file 1475-2859-10-30-S1.PNG]

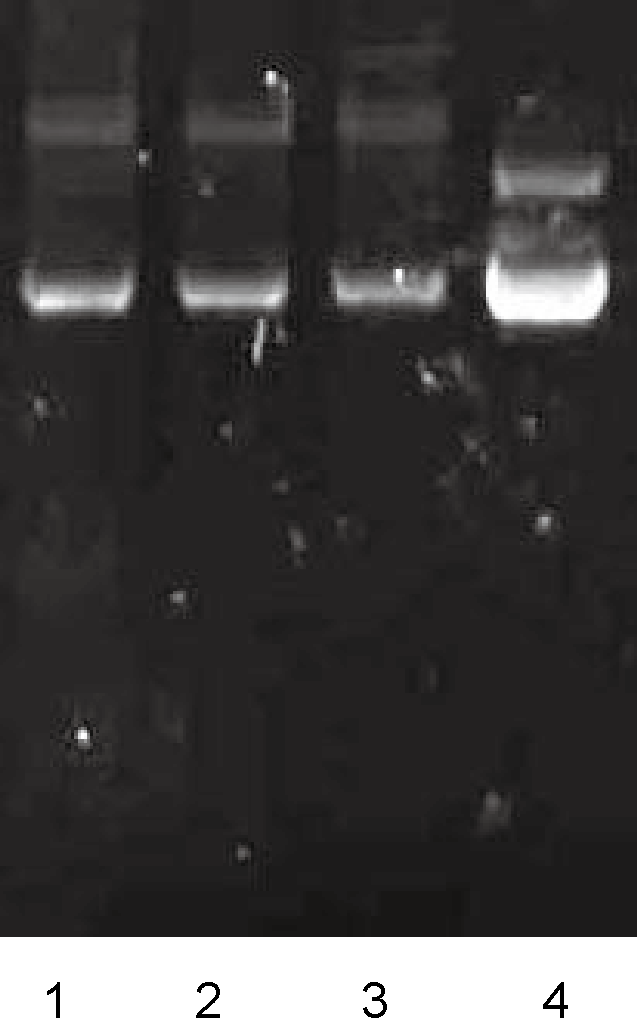

Supplement: Additional file 2 — Figure S2: Agrose gel electrophoresis of plasmid pWD. Lanes 1, 2 and 3 show the plasmid pWD isolated from transformed SM20429; Lane 4 shows the original plasmid pWD isolated from transformed E. coli. [file 1475-2859-10-30-S2.PNG]
