# Supplementary material for: Characterization of a cryptic plasmid pSM429 and its application for heterologous expression in psychrophilic Pseudoalteromonas
Source: Microb Cell Fact. 2011 May 5;10:30. doi: 10.1186/1475-2859-10-30 (PMC3112385; doi:10.1186/1475-2859-10-30)
Supplement: Additional file 3 — Characteristics of the cellulase Cel308 from the psychrophilic bacterium Pseudoalteromonas BSw20308. Purification, characterization and gene cloning of the cold-adapted cellulase Cel308 from the psychrophilic bacterium Pseudoalteromonas sp. BSw 20308. It contains Figure S3 and Figure S4. [file 1475-2859-10-30-S3.PDF]

Additional file 4: Characteristics of the cellulase from the psychrophilic bacterium *Pseudoalteromonas* BSw20308.

Purification, characterization and gene cloning of the cold-adapted cellulase Cel308 from the psychrophilic bacterium *Pseudoalteromonas* sp. BSw20308 have been performed by Dr. Pingyi Li in our lab, which have not been published. The psychrophilic strain *Pseudoalteromonas* sp. BSw20308 from the Arctic sea ice was screened for its extracellular cellulase activity. A cellulase named Cel308 was purified from strain BSw 20308 by ammonium sulfate precipitation and DEAE-Sepharose Fast Flow chromatography (Fig. S3). Effect of temperature on the activity and stability of Cel308 was measured (Fig. S4). The optimum temperature for Cel308 activity was 35°C. Cel308 had low thermostability at moderate temperatures. The half time of Cel308 activity at 45°C was about 10 min. These results indicated that Cel308 is a cold-adapted enzyme.

In order to clone the gene encoding Cel308, the N-terminal sequence of Cel308 was analyzed by Edman degradation. Based on the N-terminal sequence of Cel308, the gene encoding Cel308 was cloned by PCR and TAIL PCR from the genomic DNA of strain BSw20308. The sequence of this gene has been deposited into GenBank under the Accession No. HQ997897. Sequence analysis showed that this gene has 1479 bp corresponding to a pre-protein of cellulase with 492 amino acid residues. Domain analysis of the precursor of cellulase Cel308 indicated that it is composed of four regions: a signal peptide, an N-terminal catalytic domain belonging to glycoside hydrolase family 5, a C-terminal cellulose-binding domain belonging to carbohydrate-binding module family 5 and a linker of 107 residues connecting the two domains. As shown in Fig. S3, the mature form of cel308 only contains the catalytic domain.

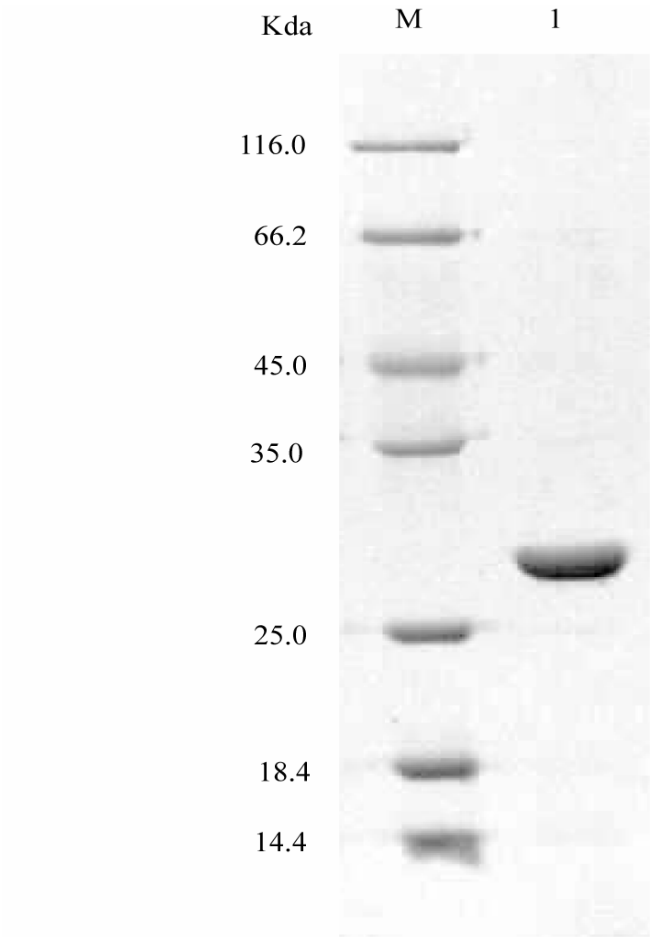

Fig. S3. The cellulase Cel308 purified from the culture of *Pseudoalteromonas* sp. BSw20308.

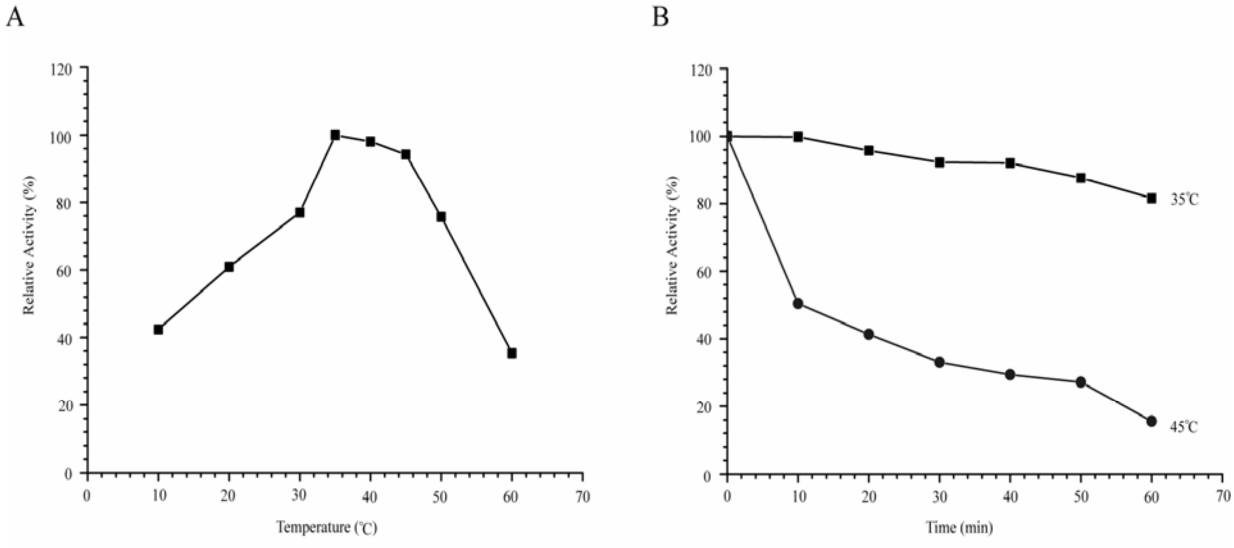

Fig. S4. Effect of temperature on the activity (A) and stability (B) of Cel308. The cellulase activity was measured as described in Materials and methods. The thermostability of Cel308 was determined by measuring the residual activity of Cel308 after incubation at 35°C or 45°C for different periods of time.
